# Systemic Granulomatosis in the Meagre *Argyrosomus regius*: Fishing for a Plausible Etiology

**DOI:** 10.3390/vetsci11120597

**Published:** 2024-11-26

**Authors:** Claudio Murgia, Tiziana Cubeddu, Giovanni P. Burrai, Alberto Alberti, Luigi Bertolotti, Barbara Colitti, Marino Prearo, Paolo Pastorino, Giuseppe Esposito, Luciana Mandrioli, Gaspare Barbera, Marina Antonella Sanna, Marta Polinas, Esteban Soto, Elisabetta Antuofermo

**Affiliations:** 1 Department of Veterinary Medicine, University of Sassari, 07100 Sassari, Italy; c.murgia3@studenti.uniss.it (C.M.); tcubeddu@uniss.it (T.C.); alberti@uniss.it (A.A.); msanna@uniss.it (M.A.S.); mpolinas@uniss.it (M.P.); eantuofermo@uniss.it (E.A.); 2 Mediterranean Center for Disease Control, 07100 Sassari, Italy; 3 Department of Veterinary Sciences, University of Turin, 10095 Grugliasco, Italy; 4 Istituto Zooprofilattico Sperimentale del Piemonte, Liguria, e Valle d’Aosta, 10154 Turin, Italy; marino.prearo@izsto.it (M.P.); paolo.pastorino@izsto.it (P.P.); giuseppe.esposito@izsto.it (G.E.); 5 Department of Veterinary Medical Sciences, Alma Mater Studiorum, University of Bologna, 47042 Cesenatico, Italy; luciana.mandrioli@unibo.it; 6 Gruppo Riservazzurra S.r.l., Cabras, 09072 Oristano, Italy; 7 Department of Medicine & Epidemiology, School of Veterinary Medicine, University of California, Davis, CA 95616, USA; sotomartinez@ucdavis.edu

**Keywords:** *Mycobacterium chelonae*, granulomas, in situ hybridization assay, fish aquaculture

## Abstract

Meagre (*Argyrosomus regius*) is one of the fast-growing species considered for sustainable aquaculture development along the Mediterranean and Eastern Atlantic coasts. The emergence of Systemic Granulomatosis (SG), a disease marked by multiple granulomas in various tissues, poses a significant challenge in meagre aquaculture. In the current study, we investigate the association of *Mycobacterium* spp. and SG in offshore aquaculture facilities in Sardinia, Italy. A total of 34 adult seemingly healthy meagre were arbitrarily collected and analyzed, combining histological, microbiological, molecular, metagenomics, and in situ techniques to investigate the presence of pathogens. Ziehl–Neelsen (ZN), periodic acid–schiff (PAS), and Giemsa stains were performed for the detection of acid-fast bacteria, common parasites, and fungi within granulomas, respectively. Granulomas were detected in 91% (31/34) of fish. The affected organs were kidney (88%), liver (47%), heart (41%), intestine (17.6%), and brain (5%). Acid-fast staining, along with *Mycobacterium* spp. specific quantitative PCR (qPCR), in situ hybridization (ISH) assay, and microbiological analyses showed negative results for the detection of *Mycobacterium* spp. and other bacteria implicated in granuloma formation. However, PCR amplification and sequencing of the 65-kDa heat shock protein gene revealed the presence of *M. chelonae* in 13% of both formalin-fixed and frozen liver tissues. Bacterial isolation failed to detect nontuberculous mycobacteria (NTM) and other bacteria typically associated with granulomas. Consistently, the use of an *M. chelonae*-specific probe in ISH failed to identify this bacterial species in granulomas. Collectively, results do not support the role of *M. chelonae* in the development of granulomas and suggest rejecting the hypothesis of a potential link between NTM and SG.

## Simple Summary

Meagre (*Argyrosomus regius*) is a fast-growing species used in aquaculture, but it faces significant challenges from Systemic Granulomatosis (SG), a disease marked by granulomas in various internal organs. This study investigated the possible link between *Mycobacterium* spp. and SG in 34 seemingly healthy meagre from a Sardinian aquaculture facility. Granulomas were identified in 91% of the fish. Several diagnostic methods, including acid-fast staining, microbiology, PCR, and in situ hybridization, were employed. While *M. chelonae* was detected in a low percentage through PCR (13%), no evidence suggested its role in SG onset.

## 1. Introduction

Meagre (*Argyrosomus regius*) is one of the fast-growing species considered for sustainable aquaculture development along the Mediterranean and Eastern Atlantic coasts [1,2]. In 2022, the global production of meagre amounted to 49,723.58 tons of live weight, primarily concentrated in Europe, contributing 11,430.68 tons [3]. Despite meagre production increasing, farms face significant challenges due to systemic granulomatosis (SG), a prevalent disease of unknown etiology that hampers growth and production performance [4,5,6]. Systemic granulomatosis is a chronic condition characterized by low mortality, high prevalence and severity, and non-specific clinical signs and gross changes such as emaciation, skin erosion, and exophthalmia. The disease is marked by multifocal white nodules visible throughout the viscera, particularly in the kidney, spleen, and liver [4]. Microscopically, the nodules correspond to granulomas consisting of concentric layers of epithelioid cells around a necrotic core and surrounded by spindle cells, which may become calcified over time or merge to form larger inflammatory foci as the condition progresses [4,5,7,8,9].

Although SG is a disease of unknown etiology, it is suggested to be linked to either a nutritional deficiency or an infectious agent [4,5,6,9,10,11,12,13,14]. Specifically, Ruiz and co-authors [12,13,15] investigated how nutritional factors, particularly antioxidants, such as vitamins E and C, and variations in diet composition influenced the incidence of SG and granuloma development in meagres. *Artemia* spp.-fed larvae were found to decrease the incidence of granulomas and reduce thiobarbituric acid-reactive substances (TBARS) levels, which improved growth and survival. Furthermore, an association between SG and larval oxidative status was identified, with diets high in docosahexaenoic acid (DHA) and low in vitamin E, correlating with a higher frequency of granulomas and increased lipid peroxidation in meagre larvae [12,13,15]. Recent findings revealed a higher incidence of granulomas in meagre fed with lower concentrations of long-chain polyunsaturated fatty acids [14]. Furthermore, Pavloudi and co-authors [9] conducted metagenomic analyses on meagre kidney tissues revealing distinct microbial profiles in healthy and granuloma-affected tissues, identifying specific operational taxonomic units (OTUs) enriched in diseased fish, though none were linked to known granuloma-causing species [9]. On the other hand, *Photobacterium damselae* subsp. *piscicida* [16,17] and *Nocardia* spp. have been reported in diseased meagre [18,19], and their role as the etiological agent of SG has been investigated [6]. Several other bacterial species have been associated with granulomas in fish [8], and nontuberculous mycobacteria (NTM) have been frequently reported in aquaculture outbreaks in the Mediterranean Sea [20,21,22]. Increases in water temperature, coupled with high stocking densities typical of intensive marine cage aquaculture, are suspected to increase the frequency and intensity of disease outbreaks, including piscine mycobacteriosis [23,24].

From a histological point of view, the meagre’s SG mirrors mycobacteriosis in fish, a systemic infectious disease caused by NTM that leads to the formation of multiple granulomas, particularly in the kidney, liver, and spleen [8,25]. Mycobacteriosis caused by *Mycobacterium marinum* infections was first described in farmed meagre in Turkey in 2014, with subsequent cases documented in the same region in 2015 [10,11]. More recently, *M. pseudoshottsii* was isolated from a meagre presenting granulomas in a Greek aquaculture farm [26]. Several NTM mycobacterial species have been associated with mycobacteriosis in fish, including *M. marinum*, *M. chelonae*, and *M. fortuitum*, as well as other species such as *M. shottsii*, *M. pseudoshottsii*, *M. ulcerans*, *M. abscessus*, *M. salmoniphilum*, *M. haemophilum*, and *M. gordonae* [25,27,28]. In particular, *M. chelonae* was identified in a wide range of aquatic environments, affecting both freshwater and marine fish species, including salmonids [29,30], sturgeons [31], mugilidae [32], and several ornamental species [33]. Isolation of Mycobacteria may be challenging due to the fastidious nature and slow growth of *Mycobacterium* spp. and their peculiar cell wall [25]. Isolation typically requires nutrient-rich and sometimes selective media such as Löwenstein–Jensen and Stonebrink. Colonies can take 2 to 28 days to form, sometimes requiring 2 to 3 months of incubation to rule out infection. Histologically, most mycobacteria are regarded as acid-fast positive, typically stained with Ziehl–Neelsen (ZN) or Fite–Faraco stains; however, depending on species and bacterial load, acid-fast negative granulomas can be found. Molecular diagnostic methods typically target different Mycobacteria conserved regions of the heat shock protein 65kD gene (*hsp65*), the exported repeated protein (*erp*), the RNA polymerase B subunit (*rpoB*), and others [25]. The detection of pathogenic agents, such as acid-fast mycobacteria within granulomas, can sometimes be challenging [34,35]. Nevertheless, the presence of NTM and other etiologic agents within affected tissues by PCR alone does not necessarily prove a link between agents and diseases. To solve this problem, in situ hybridization (ISH) techniques have been largely used in mammals, and recently, ISH has emerged as a powerful tool to detect and localize specific RNA or DNA sequences within fish tissues [36,37,38].

This study aimed to explore a mycobacteria etiology in systemic granulomatosis by using 34 adult meagres from an offshore aquaculture facility in Sardinia, Italy. Histological, metagenomic, microbiological, and molecular analyses, including in situ hybridization, were conducted to assess any possible link between SG-granulomas and NTM, in particular, *M. chelonae*.

## 2. Materials and Methods

### 2.1. Sample Collection and Gross Examination

In June 2022, 34 seemingly healthy adult meagres (*Argyrosomus regius*), except for 5 showing exophthalmia and skin hyperemia, were collected from an offshore aquaculture facility located in Sardinia, Italy. The facility maintained a stocking density of 10–20 kg/m^3^, with water salinity levels between 37 and 40‰ and a surface temperature of 25 ± 1.0 °C. The adult fish averaged 34.5 ± 3.5 cm in total length and 389 ± 67 g in total weight. Fish were euthanized by an overdose of tricaine methanesulfonate MS222 (MS-222, Sigma-Aldrich, Milan, Italy).

Necropsies of fish were performed at the Department of Veterinary Medicine, Sassari University, and aliquots of fish tissues (i.e., brain, heart, liver, spleen, kidney, and intestine) were fresh-frozen (FrFr) at −80 °C for molecular and microbiological analysis or fixed in 10% buffered formalin (Bio-Optica, Milan, Italy) for 48 h for histopathology.

### 2.2. Histopathology

For histopathology, 34 adult meagre formalin-fixed tissues were dehydrated with increasing alcohol concentrations and xylene in an automatic tissue processor and paraffin-embedded. Thin sections (3 µm) were then obtained from the formalin-fixed paraffin-embedded (FFPE) tissue blocks with a microtome (RM2245, Leica Biosystems, Wetzlar, Germany) and stained with hematoxylin and eosin (HE) in an automatic multistainer (ST5020, Leica Biosystems, Wetzlar, Germany). Slides were then evaluated at light microscopy (Nikon Eclipse 80i, Amsterdam, The Netherlands). Additionally, ZN, PAS, and Giemsa staining was performed to rule out the occurrence of acid-fast organisms, fungi, or parasites [39].

### 2.3. Metagenomic Analysis

Based on histopathological analyses confirming the presence of evident granulomas in multiple organs, such as the brain, heart, spleen, kidney, and intestine tissues of a meagre, were tested by 16S ribosomal rRNA PCR and sequencing [40].

Briefly, DNA was extracted with a DNeasy Blood and Tissue Kit (Qiagen, Milan, Italy), amplified as described, treated with Thermolabile Exonuclease I (NEB), and finally, used in Illumina Nextera XT indexing. Afterward, sample DNA concentrations were normalized to equal concentrations. Paired-end sequencing was performed on the MiSeq platform (Illumina, San Diego, CA, USA) with the v3-600 cycle chemistry.

Sequencing data were analyzed as previously reported using the operational taxonomic unit (OTU) approach, with 97% sequence similarity, using the Qiime2 package v. 2019.10 and Greengenes v. 13.8 databases [41,42]. Graphical representations were created using GraphPad Prism (version 8.0.2, GraphPad Software, La Jolla, CA, USA).

### 2.4. Microbiological Analysis and Nontuberculous Mycobacterial (NTM) Culture Screening

Microbiological analysis was performed on the kidneys of 33 out of the 34 meagre. Aseptically collected kidneys were initially homogenized in 0.8% NaCl solution, and a sterile loop with ~10 µL was inoculated in Columbia Blood Agar (CBA) and Tryptic Soy Agar (TSA) supplemented with 2% NaCl as the primary isolation media [21,43]. Plates were incubated at 22 ± 2 °C for a total of 72 h, with daily checks for bacterial growth. 

Additionally, liver, brain, heart, spleen, and intestine tissues from 33 out of the 34 meagres were individually suspended in a physiological solution and homogenized using a Seward™ Stomacher™ Model 400 circulator lab blender (Thermo Fisher Scientific, Waltham, MA, USA). Following decontamination with a 1.5% solution of 1-hexadecylpyridinium chloride monohydrate (Thermo Scientific, Milan, Italy) for 30 min, the homogenates were centrifugated at 3000 rpm for 20 min. A 10 μL pellet was then inoculated onto Löwenstein–Jensen medium and Stonebrink medium (Microbiol, Uta-Cagliari, Italy) tubes using a sterile loop. Two tubes were prepared for each medium; one set was incubated for 60 days at 28 ± 1 °C and the other at 37 ± 1 °C, with daily monitoring. The colonies were stained using the Ziehl–Neelsen technique with cold-modified carbolfuchsin (Kinyoun staining) [44]. The meagre that was submitted to metagenomic analysis did not undergo microbiology. 

### 2.5. Molecular Identification of Mycobacterium spp.

#### 2.5.1. DNA Extraction

DNA extraction was performed from 34/34 FFPE and FrFr livers of meagre with SG and 30/34 FrFr kidneys with SG. Based on histology, 3/34 meagre did not show granulomas, and livers from these fish were used as negative controls. For the FFPE tissues, 15 sections 3 μm in thickness were obtained with a Leica RM2245 microtome (Leica Biosystems, Nussloch, Germany) and placed into 1.5-mL sterile microtubes. To avoid cross-contamination, the samples were collected using disposable gloves and masks. The blades were changed for each FFPE block, while the microtome, instruments, and all work surfaces were cleaned with 70% ethanol. The sections were then deparaffinized using xylene. Briefly, the sections were submerged in 1 mL of xylene vortexed and centrifuged at 10,000× *g* for 10 min. The xylene was carefully removed, and the tissue was washed twice with 100% ethanol to remove any residual xylene and centrifuged at 14,000× *g* for 5 min to pellet the tissue. Following the ethanol washes, the tissue pellets were air-dried at 37 °C for 15–20 min to evaporate any remaining ethanol and subjected to DNA extraction using a QIAGEN DNEasy Blood and Tissue kit according to the manufacturer’s instructions. For the 30 FrFr kidneys, DNA extraction was also performed using the QIAGEN DNEasy Blood and Tissue kit, following the manufacturer’s instructions (Qiagen, Milan, Italy). DNA concentration and purity were assessed using a NanoDrop ND-2000 (Thermo Scientific, Milan, Italy). 

#### 2.5.2. Polymerase Chain Reaction (PCR)

Molecular identification and characterization of pathogens were conducted using PCR amplification and sequencing of the 65-kDa heat shock protein (*hsp65*) gene on both 34/34 FFPE and FrFr livers. Meagres without granulomas (3/34) were used as negative controls. The amplification of a ~441 bp fragment of the *hsp65* gene was carried out, according to Telenti et al. [45]. The PCR mix contained 1 × reaction buffer (with 1.5 mM MgCl2), 1 × CoralLoad, 200 mM dNTP, 25 pmoles of each primer, 1U TopTaq DNA polymerase (Qiagen, Milan, Italy), and 5 µL of total DNA in 50 µL of reaction. PCR amplification consisted of an initial denaturation at 93 °C for 10 min, followed by 45 cycles of 94 °C for 1 min, 60 °C for 1 min, and 72 °C for 1 min, with a final extension at 72 °C for 7 min. PCR products were analyzed by 2% agarose gel electrophoresis, and fragments were detected using GelRed staining and a UV transilluminator. *Mycobacterium marinum* (DSM 44344) was used as a positive control. DNA isolated from meagres without granulomas and DEPC-treated H_2_O were used as negative controls.

The positive PCR products were gel-purified using the QIAquick Gel Extraction Kit (Qiagen, Milan, Italy) and confirmed by sequencing on an ABPRISM 3500 Genetic Analyzer (Applied Biosystem, Monza, Italy). The electropherograms were manually edited for sequence accuracy using BioEdit v. 7.2.5 (Hall, 1999). Subsequently, the sequences were queried against GenBank’s Basic Local Alignment and Search Tool (BLAST) at https://www.ncbi.nlm.nih.gov/ to identify closely related sequences (accessed on 5 December 2023). For phylogenetic analysis, MEGA v.7 software was employed [46]. The phylogenetic tree was constructed using the maximum-likelihood method, and evolutionary distances were calculated utilizing the Kimura 2-parameter model with 1000 bootstrap replicates. 

#### 2.5.3. Real-Time Quantitative PCR (qPCR)

Detection and quantification of *Mycobacterium* spp. DNA was performed on 30/34 FrFr kidneys from SG-affected meagre samples using qPCR, targeting the genus-specific *Mycobacterium atpE* gene, according to Radomski et al. [47]. Samples that underwent metagenomic and kidneys from meagre without granulomas were not included. The 12 μL reactions consisted of 6 μL TaqMan Environmental Master Mix 2.0 (Applied Biosystems, Monza, Italy), 0.25 μM of each primer and 0,05 μM of the probe, 5 μL of template DNA, and RNase-free water adjusted to volume. *Mycobacterium chelonae* (DSM 43804) was used as a positive control, and DEPC-treated H_2_O was used as a negative control. All samples, including positive and negative controls, were run in triplicate. Cycling conditions were as follows: 30 s at 60 °C; 10 min at 95 °C; 40 cycles of 15 s at 95 °C (denaturation); 60 s at 60 °C for the annealing; and 30 s at 60 °C of final extension. All qPCRs were analyzed using the QuantStudio3 qPCR System (Thermo Scientific, Milan, Italy). The cycle threshold line was set at 0.05 ΔRn units; Cq values above 36 were considered negative. All primers used for molecular analysis are listed in Table 1.

### 2.6. In Situ Hybridization Assay

Manual RNAscope assay was set up and performed at the Department of Veterinary Medicine University of Sassari on FFPE of the 30/34 SG-affected meagre using BaseScopeTM v2 Assay (cod. # 322350, Bio-Techne, Milan, Italy), according to the manufacturer’s protocol [48]. Briefly, after deparaffinization and rehydration steps, tissues were immersed in a solution of 1% SDS containing 200 mM of boric acid, pH 7.0, at room temperature, followed by ½ hour at 37 °C in the same solution. Then, the slides were washed 3 times with 0.2% Tween-20—PBS. Tissue sections were treated with RNAscope^®^ Hydrogen Peroxide (Bio-techne, Milan, Italy) for 10 min at room temperature. Target retrieval was performed for 15 min at 100–104 °C. Probes were then hybridized for 2 h at 40 °C, followed by RNAscope amplification and red chromogenic detection. Sections were counterstained with hematoxylin and mounted with Bio-Mount (Bio-Techne, Milan, Italy).

In this study, the following RNAscope probes were used: probe encodes for B-*Mycobacterium chelonae*-16SrRNA targeting 169–209 of DQ866772.1. (cod. # 1301541-C1, Bio-Techne, Milan, Italy) and dihydrodipi–colinate reductase (dapB), a bacterial gene (cod. #310043, Bio-Techne, Milan, Italy), as a negative control probe. 

As a positive control of the probe encodes for B-*M. chelonae*-16SrRNA targeting 169–209 of DQ866772.1, an FFPE goldfish *Carassius auratus* block experimentally infected with *M. chelonae*, kindly provided by the Aquatic Animal Health Laboratory at the School of Veterinary Medicine, University of California, Davis, was used.

## 3. Results

### 3.1. Gross Examination

**Figure 1:**
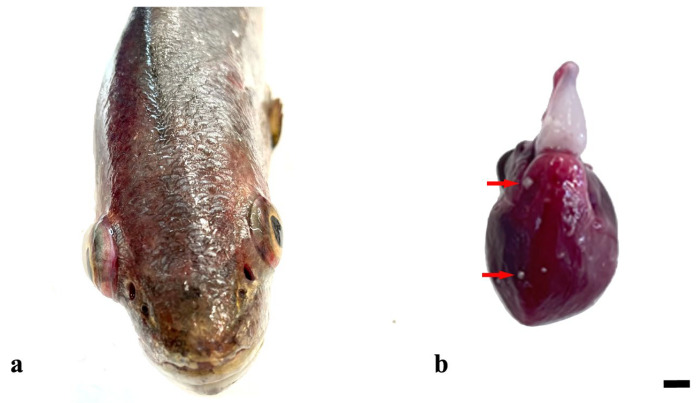
(**a**) Affected meagre showing severe hemorrhages and bilateral exophthalmia. (**b**) Heart of meagre with visible white nodules (red arrows) on the epicardium. Bar: 0.2 cm.

At gross examination, exophthalmia and skin hyperemia were observed in 5/34 (15%) meagres (Figure 1a), whereas 1/34 (2%) exhibited macroscopic multifocal white, slide raised, 0.2 × 0.2 cm ovoidal nodules both in the posterior kidney and in the heart (Figure 1b).

### 3.2. Histopathology

**Figure 2:**
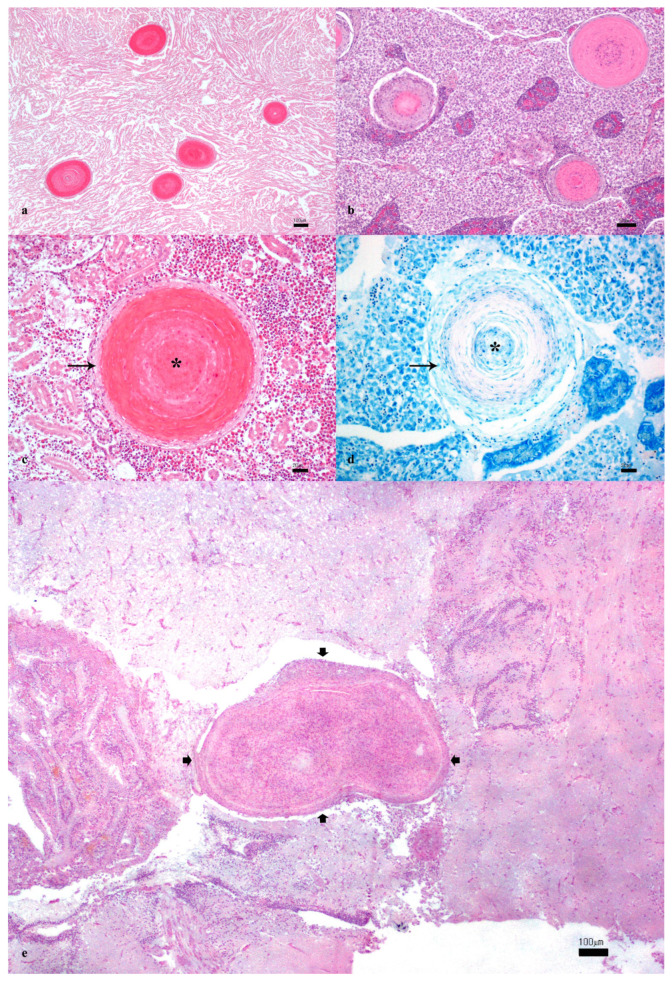
(**a**) Multifocal granulomas in the heart (Hematoxylin and eosin—H&E. Bar: 100 µm). (**b**) Multifocal granulomas in the liver (H&E. Bar: 100 µm). (**c**) High power field of a granuloma in the kidney characterized by a necrotic hypereosinophilic center (asterisk) surrounded by epithelioid and spindle cells arranged in concentric layers (arrows) (H&E. Bar: 20 µm). (**d**) Negative Ziehl–Neelsen stain of a granuloma in the kidney (ZN. Bar: 20 µm). (**e**) Focal granuloma in the brain (thick arrows) (H&E. Bar: 100 µm).

Histopathological examination revealed the presence of multifocal granulomas in 31/34 (91%) adult meagres. The kidney was the most affected organ (30/34; 88%), followed by the liver (16/34; 47%), the heart (14/34; 41%), the intestine (6/34; 18%), and the brain (2/34; 6%) (Figure 2a–e). Notably, 3/34 (9%) meagre specimens were negative for granulomas in all examined organs and were considered non-SG-affected fish.

Microscopically, the parenchyma was characterized by a severe granulomatous inflammation with the presence of multiple 200 to 900 microns of diameter nodules. Granulomas were characterized by a central region rich in abundant eosinophilic granular material, intermixed with numerous cellular debris (lytic necrosis) and surrounded by multiple layers of epithelioid cells and a rim of elongated spindle cells (Figure 2c). No lymphoplasmacytic inflammation was noticed. ZN staining (Figure 2d), as well as PAS and Giemsa, tested negative in all evaluated organs and sections.

### 3.3. Metagenomic Analysis

The metagenomic analysis, along with unclassified species, showed that the most predominant phyla were Proteobacteria (88.6% for the intestine, 24.1% for the brain, 37.2% for the heart, 14.1% for the kidneys) and Bacteroidetes (46.8% for the spleen), followed by Firmicutes (31.6% for the heart, 12.3% for the kidneys, 9.4% for the brain, 7.7% for the intestine, 31.9% for the spleen). In the phylum Proteobacteria, the most predominant class was Gammaproteobacteria (43.9% for the intestine, 33.4% for the heart, 18.9% for the brain, 11.2% for the kidneys, 8.2% for the spleen), with the most abundant species *Sulfitobacter donghicola* (41.7%) for the intestine, *Faecalibacterium prausnitzii* (4.9%) for the kidney, *Acinetobacter johnsonii* (18.2%) for the heart, and *Veillonella dispar* (0.6%) in the spleen.

**Figure 3:**
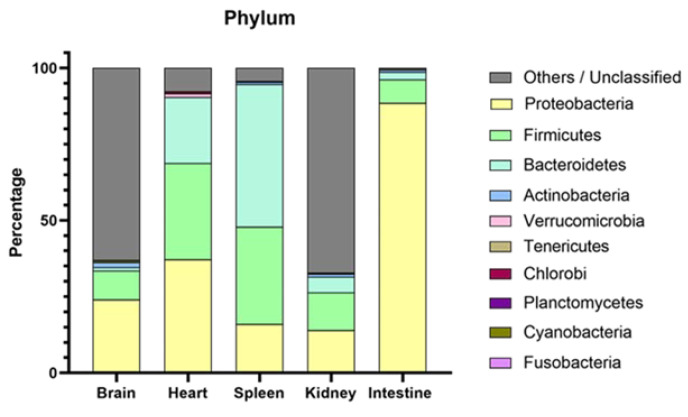
Bar chart illustrating the relative abundance (in percentage) of the main microbial taxa at the phylum level in the brain, heart, spleen, kidney, and intestine of a meagre affected by systemic granulomatosis.

In the phylum Firmicutes, the most predominant classes were Bacilli (31.5% in the heart), Alphaproteobacteria (41.7% in the spleen and 7.7% in the intestine), and Clostridia (10.1% in the kidney and 7.8% in the brain). The most abundant species were *Acinetobacter lwoffii* (12.9%) in the heart, *Chitinibacter tainanensis* (1.4%) across various organs, and *Bacteroides uniformis* (0.6%) in the kidney. For Bacteroidetes, Bacteroidia was the class with the most reads for the spleen with the most abundant species, *Faecalibacterium prausnitzii* (11.6%) (Figure 3).

### 3.4. Microbiological Analysis

No bacteria were recovered in the inoculated Columbia Blood Agar, TSA (2% NaCl) in Löwenstein–Jensen or Stonebrink medium.

### 3.5. Molecular Detection and Identification of Mycobacterium spp.

**Figure 4:**
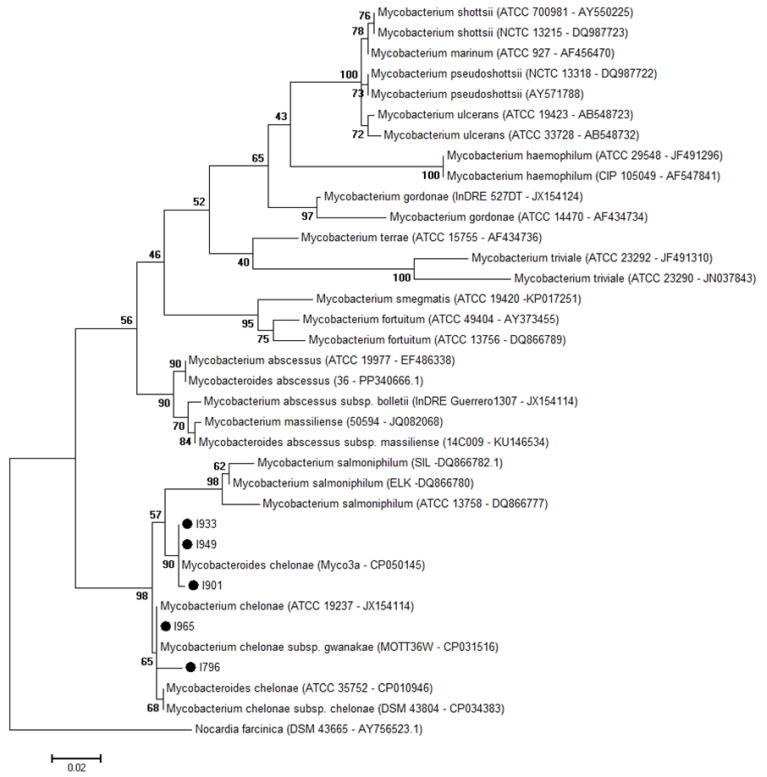
Maximum Likelihood tree shows the hsp65 sequences obtained from systemic granulomatosis-affected meagre cluster with the *Mycobacterium chelonae*. *Nocardia farcinica* was selected as the outgroup. The other sequences are the most probable species causing mycobacteriosis in fish. Evolutionary analyses were conducted in MEGA7, performed using Maximum Likelihood method, Tamura–Nei model, and bootstrap 1000 replicates.

From PCR analysis, 7/34 (20%) FFPE livers, as well as 6/34 (18%) FrFr livers, showed a ~440 bp band. The PCR products were purified and sequenced, and electropherograms were edited for sequence accuracy and were aligned. *Mycobacterium chelonae* was detected in 5/31 (16%) SG-affected meagre and, in particular, in 4/31(13%) FFPE livers and in 4/31(13%) livers FrFr of SG-affected meagre (Table 2; Figure 4). Mycobacteria sequences share 99–100% nucleotide identity with hsp65 of *Mycobacterium chelonae* strains available on the GenBank database. The derived sequences were submitted to GenBank, and their accession numbers were PQ340275-PQ340276-PQ340277-PQ340278-PQ340279. The *atpE* qPCR results were negative for *Mycobacterium* spp. detection in all the tested kidneys.

### 3.6. Association Between Histology and Mycobacteriun chelonae

In 16/31 (52%) livers showing granulomas from meagre with SG, M. chelonae was detected in 4/16 (25%) samples, whereas M. chelonae was not identified in 15/31 (48%) in non-affected livers, both FFPE and FrFr, except for one case (1/15; 0.6%) (Table 2).

### 3.7. In Situ Hybridization Assay

**Figure 5:**
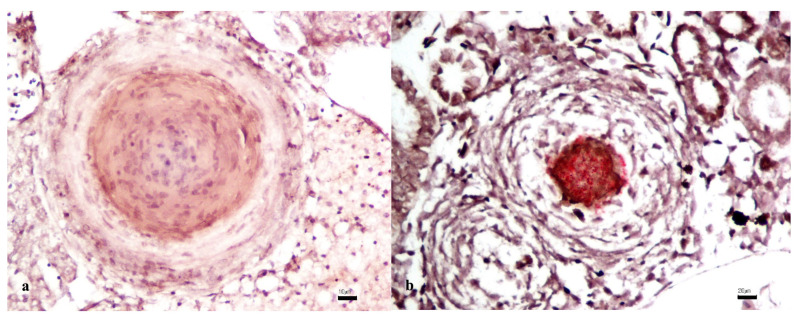
(**a**) Granuloma in meagre’s liver without in situ hybridization signals (Hematoxylin counterstain. Bar: 20 µm). (**b**) Numerous, 1–2 microns in length, bacillary red rods (red chromogen) inside a granuloma of a *Carassius auratus* experimentally infected with *M. chelonae* (Hematoxylin counterstain. Bar: 20 µm).

No positive ISH signals were observed in any of the meagre’s evaluated organs, whereas in the control tissues of the *Carassius auratus* experimentally infected with *M. chelonae,* numerous, 1–2 micron in length, bacillary rods inside a granuloma were observed (Figure 5a,b). 

A summary of the results obtained by histopathological examination, microbiology, PCR, and ISH is presented in Table 3.

## 4. Discussion

Granulomatous diseases in fish are commonly associated with chronic infections and inflammatory responses to persistent antigens, often caused by bacteria, fungi, or noninfectious agents. In meagre, a valuable aquaculture species, systemic granulomatosis (SG) presents as widespread granulomas across multiple organs, posing significant challenges for diagnosis and management and prompting this investigation into its underlying causes and characteristics. Thus, this study focused on describing gross findings, histopathology, metagenomic analysis, microbiology, and molecular identification of SG disease in meagre. While this study was limited to meagre sampled from a single farm at one time, this controlled approach minimized confounding variables such as environmental factors (e.g., water quality, temperature, and salinity) that might arise from multiple farms or sampling periods, enabling a more focused analysis of SG etiology and manifestation in meagre. 

Gross examination revealed exophthalmia and skin hyperemia in 5 out of 34 meagres. Similar aspecific signs have been reported in various fish species suffering from systemic infections, including those caused by *Mycobacterium* spp. and other pathogens [25]. Histopathological analysis further confirmed the presence of granulomas characterized by a central region of eosinophilic granular material and cellular debris, surrounded by multiple layers of epithelioid cells and spindle cells in different organs, with the kidney being the most affected (30/34), followed by the liver (16/34) and heart (14/34). Granulomas in the kidney, liver, and heart align with findings in other studies on fish granulomatous diseases, where these organs are commonly affected [7]. Furthermore, while the kidney is often reported as a primary site of granulomatous inflammation, the prevalence of granulomas in the heart and liver observed in this study is relatively high compared to some reports, which may suggest species-specific responses or environmental factors influencing disease manifestation [27,49,50,51]. Likewise, as previously described by other authors, no acid-fast bacteria, fungi, or parasites were observed within the granulomas in our study using histological staining, including ZN [4,5,6,10,11,12,13]. Granuloma’s pathogenesis is variable and of a complex nature, and granuloma can be defined as a localized inflammation or a hypersensitive reaction to substances leading to different layers of macrophages [27]. Etiologies of granulomatous disorders can be divided into infectious and noninfectious causes (autoimmune conditions, toxins, body reactions, etc.) [52]. In some cases, it is hard to distinguish infectious from noninfectious granulomas. However, pathologists agree that when granulomas exhibit a necrotic “core”, extracellular bacteria can persist, surrounded by cellular necrotic debris. Accordingly, in our study, the histopathological pattern of the disease with the necrosis centrally located in meagre’s granulomas directs the investigations toward identifying target pathogen-causing granulomas in fish [52]. 

Metagenomic analyses revealed the prevalence of various bacterial phyla in different organs, reflecting the complex microbiota associated with granulomatous inflammation. Bacteroidetes were predominant in the spleen, while Proteobacteria were more common in other organs. Firmicutes were also significantly present, whereas Actinobacteria had a low prevalence in the kidney, the most affected organ. Of note, the low prevalence of Actinobacteria, particularly *Mycobacterium* spp., despite their known pathogenicity, would decrease their likelihood as a cause of SG [53,54]. These data do not differ from what has been recently proposed by Pavloudi et al. [9], suggesting that a “new” pathogenic bacteria species or a single predominant pathogen was not present in SG. Although their specific roles remain to be elucidated, this diverse bacterial community might contribute to the observed granulomatous inflammation, and their presence in significant proportions implies potential interactions with the host immune system that may exacerbate inflammation. Although the metagenomic analysis was performed on a representative case of meagre affected by SG, involving multiple organs, including the brain, it is important to acknowledge the inherent limitations of using a single case for such studies. Furthermore, culture, which remains the gold standard in cases of bacteria-causing diseases, tested negative for bacteria and mycobacteria in our work. The absence of cultivable bacteria might indicate that the granulomas had a non-bacterial etiology. However, many pathogens, including certain species of *Mycobacterium*, exhibit stringent growth requirements or are slow-growing, which may not be met by conventional culture media [25,55]. Overall, culture supports the hypothesis that mycobacteria were not the causative agents of the granulomas, although granulomas without visible acid-fast bacilli have been reported in several experimentally infected species [53,56,57]. Molecular diagnostic methods are now considered the most practical and efficient approach to detecting and identifying mycobacteria at the species level, mainly overcoming the limitations of culture and biochemical tests [58]. These PCR methods may involve the amplification of DNA from fresh, frozen, or ethanol-preserved tissues and often target highly discriminatory regions, such as *hsp65*, *rpoB*, and the 16S–23S internal transcriber spacer [25,59,60]. In our study, PCR targeting the *hsp65* gene of mycobacteria yielded positive results in a subset of FFPE and FrFr liver samples, with sequencing identifying *M. chelonae* in 4/34 FFPE and FrFr liver samples. 

Mycobacterial infections in fish are frequently reported in both wild and farmed species across marine and freshwater environments [27,56,61,62,63]. Less frequent cases of systemic mycobacteriosis caused by *M. marinum* were reported in the meagre [10,11]. However, to our knowledge, *M. chelonae* has not been previously identified in farmed meagres showing systemic granulomas. On the other hand, in our study, the qPCR analysis targeting the *atpE* gene for *Mycobacterium* spp. quantification in kidney samples tested negative, not supporting the SG infection caused by NTM (atypical mycobacteria, as reported by Avsever et al. [10] and Timur et al. [11].

Previous studies have compared methods of detecting mycobacteria in fish tissues, including histological and bacteriological examination and nucleic acid-based techniques [64,65]. However, Gauthier and co-authors [53] pointed out several drawbacks of different detection methods due to their focus on varying biological phenomena. Histological methods can identify disease and infection but are not powerful in cryptic infections in which the pathogen is undetectable in the histological sections and is not cultivable. Bacteriological examinations detect culturable organisms, indicating the presence of live bacteria capable of multiplying and infecting new hosts. However, as suggested by Pourahmad and co-authors [66], a robust immune response in fish might resolve an active mycobacterial infection, rendering the bacteria nonviable and unculturable. Conversely, molecular methods detect mycobacterial DNA without confirming bacterial viability or disease presence. In such cases, significant amounts of mycobacterial DNA might still be detected by PCR, indicating mycobacteriosis even if the bacteria are no longer viable [66]. Overall, different scenarios are, therefore, possible, including infection due to some cryptic bacteria, a state of non-infection but with mycobacterial DNA present within affected tissue, as well as granulomas caused by noninfectious agents [53].

In addition to SG, a well-known condition in fish pathology named visceral granulomatosis exists as an aspecific tissue response to numerous aetiologic agents, of which Mycobacteria are most recognized and described in the literature; in the study about doctor fish [62], granulomas were detected in all of the batches analyzed, and *Mycobacterium* antigens have been immunohistochemically (IHC) labeled, confirming their presence within pathologic tissue, stressing the relevance to flank an in situ technique as IHC to histochemistry and/or molecular analyses, especially in research or from the field studies. Moreover, the diagnostic challenge of mycobacteria in the case of granulomas containing few bacilli has driven pathologists to the increased use of RNA scope. This technique is highly sensitive and specific, using a unique probe design to amplify target RNA signals, allowing for the detection and visualization of a colorimetric signal within formalin-fixed paraffin-embedded tissue samples. In this study, the decisive value of RNAscope was demonstrated on various granuloma-affected meagre organs, including the kidneys, liver, heart, and intestine, showing that mycobacterial RNA was not detected, although *M. chelonae* DNA was detectable in a few liver samples. The goal of this study was achieved in particular due to the set-up of ISH. The absence of a specific ISH signal reinforces the lack of association between mycobacteria and granulomas and confirms that there was no active infection or significant bacterial load in tissues examined in meagre affected by SG.

## 5. Conclusions

Our findings suggest that *Mycobacterium chelonae* DNA can be detected in granuloma-affected tissues of meagre; however, it is unlikely to be the primary cause of systemic granulomatosis (SG). SG is regarded as a noninfectious condition characterized by Ziehl–Neelsen negative granulomas, potentially stemming from unproven nutritional or autoimmune causes. Furthermore, its similarity to systemic mycobacteriosis caused by *M. marinum* [8,25] highlights the need for expertise in differentiating granuloma types through histopathology. Future research should focus on identifying the diagnostic features necessary to distinguish granuloma types in meagres by utilizing advanced diagnostic methods such as direct granuloma sampling, molecular techniques, next-generation sequencing, and experimental inoculation. Such efforts are essential to mitigate the economic impact of these conditions and to address the potential zoonotic implications of pathogens causing granulomas in meagre. 

## Figures and Tables

**Table 1:** Primers used in this study for the molecular analyses.

	Gene Name	Primer Name	Sequence (5′-3′)	Reference
***Metagenomic***	*16S rRNA*	Pro341F	TCGTCGGCAGCGTCAGATGTGTATAAGAGACAGCCTACGGGNBGCASCAG	[40]
		Pro805R	GTCTCGTGGGCTCGGAGATGTGTATAAGAGACAGGACTACNVGGGTATCTAATCC	
***PCR***	*Hsp65*	Tb11	ACCAACGATGGTGTGTCCAT	[45]
		Tb12	CTTGTCGAACCGCATACCCT	
***qPCR***	*atpE*	FatpE	CGGYGCCGGTATCGGYGA	[47]
		RatpE	CGAAGACGAACARSGCCAT	
		probe PatpE	ACSGTGATGAAGAACGGBGTRAA	

**Table 2:** Association between granulomas in meagre’s livers, PCR, and sequencing.

Meagres Id	Liver Granulomas	PCR hsp65 (FFPE *)	PCR hsp65 (FrFr *)	Sequencing
1	Yes	−	−	
2	Yes	**+**	**+**	***Mycobacterium chelonae***
3	Yes	+	−	*Propionibacterium*
4	Yes	+	+	*Corynebacterium*
5	Yes	−	−	
6	No	−	−	
7	No	−	−	
8	No	−	−	
9	No	−	−	
10	No	−	−	
11	Yes	−	−	
12	Yes	−	−	
13	**No**	**+**	**+**	***M. chelonae***
14	Yes	+	+	*Propionibacterium*
15	Yes	−	−	
16	No	−	−	
17	Yes	**+**	−	***M. chelonae***
18	Yes	−	−	
19	Yes	**+**	**+**	***M. chelonae***
20	Yes	−	**+**	***M. chelonae***
21	No	−	−	
22	No	−	−	
23	Yes	−	−	
24	No	−	−	
25	Yes	−	−	
26	No	−	−	
27	Yes	−	−	
28	No	−	−	
29	No	−	−	
30	No	−	−	
31	Yes	−	−	
32	No	−	−	
33	No	−	−	
34	No	−	−	

* FFPE: Formalin-fixed and paraffin-embedded tissue; FrFr: fresh-frozen.

**Table 3:** Summary of the results obtained by histopathological examination, microbiology, PCR, and in situ hybridization (ISH).

Analysis	Results	Meagre
Histopathology	Presence of granuloma	31/34
Histochemistry	Ziehl–Neelsen; positivity in granulomas	0/34
Microbiology	Bacteria and mycobacteria isolation	0/33
qPCR in kidney	atpE qPCR +	0/30
PCR FrFr livers	*Hsp65* PCR +	7/34
	*M. chelonae*	4/7
PCR FFPE livers	*Hsp65* PCR +	6/34
	*M. chelonae*	4/6
ISH	*M. chelonae +*	0/30

## Data Availability

The datasets generated during and/or analyzed during the current study are available from the corresponding author upon reasonable request.
